# Genetic Marker Discovery in Complex Traits: A Field Example on Fat Content and Composition in Pigs

**DOI:** 10.3390/ijms17122100

**Published:** 2016-12-14

**Authors:** Ramona Natacha Pena, Roger Ros-Freixedes, Marc Tor, Joan Estany

**Affiliations:** 1 Departament de Ciència Animal, Universitat de Lleida—Agrotecnio, 25198 Lleida, Spain; Roger.Ros@roslin.ed.ac.uk (R.R.-F.); mtor@ca.udl.cat (M.T.); jestany@ca.udl.cat (J.E.); 2 The Roslin Institute and Royal (Dick) School of Veterinary Studies, The University of Edinburgh, Scotland EH25 9RG, UK

**Keywords:** meat quality, intramuscular fat, candidate gene, pork, oleic acid, monounsaturated fatty acid

## Abstract

Among the large number of attributes that define pork quality, fat content and composition have attracted the attention of breeders in the recent years due to their interaction with human health and technological and sensorial properties of meat. In livestock species, fat accumulates in different depots following a temporal pattern that is also recognized in humans. Intramuscular fat deposition rate and fatty acid composition change with life. Despite indication that it might be possible to select for intramuscular fat without affecting other fat depots, to date only one depot-specific genetic marker (*PCK1* c.2456C>A) has been reported. In contrast, identification of polymorphisms related to fat composition has been more successful. For instance, our group has described a variant in the stearoyl-coA desaturase (*SCD*) gene that improves the desaturation index of fat without affecting overall fatness or growth. Identification of mutations in candidate genes can be a tedious and costly process. Genome-wide association studies can help in narrowing down the number of candidate genes by highlighting those which contribute most to the genetic variation of the trait. Results from our group and others indicate that fat content and composition are highly polygenic and that very few genes explain more than 5% of the variance of the trait. Moreover, as the complexity of the genome emerges, the role of non-coding genes and regulatory elements cannot be disregarded. Prediction of breeding values from genomic data is discussed in comparison with conventional best linear predictors of breeding values. An example based on real data is given, and the implications in phenotype prediction are discussed in detail. The benefits and limitations of using large SNP sets versus a few very informative markers as predictors of genetic merit of breeding candidates are evaluated using field data as an example.

## 1. Meat Quality as a Complex Trait

Meat quality has always been important to consumers and critical to guarantee returning customers. However, meat quality is difficult to define as it is a complex concept that includes appearance, sensorial, hygienic and nutritional attributes. In addition, a number of technological features, such as fat melting point, water holding capacity or pH, are crucial for the meat processing industry. Moreover, consumer preferences are specific to each particular market, which means that optimal values on certain attributes such as marbling score, color, or ageing flavor can vary widely across countries.

Regarding the sensory traits, the two attributes more important for meat consumers are tenderness and flavor. The former is directly related to muscle fiber morphology (total number of fibers and cross-sectional area) and, most importantly, to fiber type composition. The contractile and metabolic properties of muscle are differentiated by muscle fiber types. According to the main isoforms of myosin heavy chain (MHC) that are present, muscle fibers are classified as type I (MHC-1), type IIa (MHC-2a), type IIb (MHC-2b), and type IIx (MHC-2x). The type I and IIb fibers, also known as slow-oxidative and fast-glycolytic, respectively, represent two extreme metabolic profiles, while the type IIa and IIx fibers show intermediate properties. Muscles rich in type I fibers are darker, richer in intramyocytic lipids and have a lower glycolytic potential than type IIb fibers [1]. Thus, fresh meat quality is strongly related to fiber type composition in muscle and to the aging and proteolysis of these fibers during the muscle-to-meat transformation process (reviewed in [1]). Tenderness is also influenced by the amount of intramuscular fat (IMF) in the meat cut as infiltrated fat gives thinner myofibrils, which facilitates chewing (sensory tenderness) and reduces instrumental tenderness [2]. Fat accumulates in the muscle in the form of phospholipids, cholesterol but mostly triglycerides. These are mainly stored within intramuscular adipocytes (about 80%), but also within myocyte cytoplasm in droplets in close vicinity to mitochondria (5% to 20% of total triglycerides) [3]. Intramuscular fat is also a source of meat flavor, particularly in dry-cured products. In these products, oxidation of fat during meat maturation produces potent volatile flavor contributors that are released on heating.

Consumers are also concerned by the nutritional quality of foods. Fats of animal origin tend to be richer in saturated fatty acids (SFA) than vegetable fats. While consumers will favor meat cuts with little rind fat, a minimum amount of IMF is needed to ensure tenderness and flavor. Fatty acid composition of fat varies between muscles, and with the diet, live weight or fatness, age, sex, and genotype. In pigs and other monogastric animals, the fatty acid composition of the fat tissue (particularly of the subcutaneous fat) can be readily manipulated by modifying the proportions of dietary fatty acids, which are absorbed intact from the small intestine and incorporated directly into fat tissue. Thus, fat composition can be manipulated to make it less saturated, which is beneficial from a nutritional point of view. Intramuscular fat content and composition also have an important genetic component (h^2^ ≅ 0.4–0.7), which means animals can be selected for more favorable content and composition and bred to improve these traits in the next generation (see Section 3). However, results from genome-wide association studies (GWAS) show that these traits are clearly polygenic, being controlled by many genes some of which explain a relevant proportion of the genetic variance (see Section 4).

Thus, meat quality is a complex trait both at phenotypic and genotypic level. This review focuses on the genetic constitution of fat content and composition in pork and how novel technologies can contribute to identify markers that can be used in a pig breeding scheme. After an update on the latest advances regarding phenotype-based and marker-assisted selection, an example is given with field data from a Duroc selection company aiming at quality cured products (Section 5).

## 2. Relationship between IMF and Other Fat Depots in Pigs

Depending on the genetic type, subcutaneous fat accounts for 16%–31% of carcass weight in pigs [4]. It is the most abundant adipose tissue and for decades it has been selected against to improve the production efficiency of pork. Intramuscular fat content is also influenced by genetic type, ranging from 1.2%–2.7% in Pietrain pigs [5] to 2.7%–4.8% in Duroc pigs [6], although even within breed much wider ranges have been described [7]. IMF is the least abundant adipose tissue in the pig carcass and it has a favorable impact on meat quality, having optimal values around 1.5%–3% in fresh meat [8]. Both tissues have therefore been extensively studied. Because subcutaneous and intramuscular depots are two of the compartments in which adipose tissue is deposited throughout the body, their improvement cannot be independent from each other and from the rest of fat depots. In fact, during the growth period, mesenteric, omental, perirenal, subcutaneous, intermuscular and intramuscular fat depots accumulate fat following a temporal pattern that is also shared with other species such as humans (see below). Kouba and Sellier [9] reviewed allometric coefficients of adipose tissue in pigs. There are several studies that establish the growth rate of dissectible adipose tissues. The highest relative rate corresponds to the perirenal tissue, followed by subcutaneous adipose tissue and IMF. However, allometric studies for IMF are scarce. Davies and Pryor [10] using Large White x Landrace castrated males from 8 to 62 kg live weight reported allometric coefficients of 1.08, 1.01, 0.97 and 0.91 for perirenal, subcutaneous, intermuscular and intramuscular fat, respectively. Thus, IMF is the adipose tissue that shows the slowest relative growth in the carcass and also the latest development [11]. Nonetheless, the growth process of adipose tissue is not uniform over time. Subcutaneous adipose tissue grows mainly by hyperplasia at early stages (1–2 months of age), changing to a combination of hypertrophy and hyperplasia at 2–5 months of age, and increasing mainly by hypertrophy after that [12].

Despite sharing a common physiological basis, adipocytes from different fat depots display distinct regulation of cellular activities. Functional studies indicate that IMF cells have a delayed maturation and display differences in both metabolic and secretory functions [3]. The delay in the development of IMF could be explained by the competition between adipocytes and muscle fibers for the uptake of blood fatty acids [11]. Before 30 days of life, most lipid droplets are in the muscle fibers, and are rare in preadipocytes. This trend is reversed at later ages. Intramuscular adipocytes are much smaller than visceral or subcutaneous fat, at any time point [13]. In the three tissues, vacuole size has been linked to the expression of lipid droplet-associated proteins such as the *SNAP23* (synaptosomal-associated protein 23) gene, which is the lowest in IMF [14]. Distinct metabolic properties of IMF adipocytes include a low lipogenic potential as seen by low expression and activity of lipogenic enzymes and low incorporation of glucose into triglycerides independently of insulin levels [13]. Expression of hormone receptors and many secreted adipokines is also depressed in intramuscular adipocytes. This peculiar behavior is likely the result of the paracrine crosstalk between muscle and adipocytes. A number of muscle differentiation factors have antiadipogenic properties and delay preadipocyte terminal differentiation (reviewed in [15]). In addition, fatty acid synthesis in the fat cell is counterbalanced by their oxidation within muscle fibers. Regarding fat composition, the turnover of fatty acids in the muscle has to be taken into account. However, despite all these particularities, no IMF-specific factor has been described to date.

As a result of all the above, fatty acid composition in IMF is different from other fat depots. As shown in Table 1, IMF has a lower polyunsaturated fatty acid (PUFA) content and a higher percentage of SFA, while monounsaturated fatty acid content (MUFA) is similar in IMF and backfat. This is true both in the series of 16 and 18 carbon atoms, which represent the main fatty acids in pig fat. Oleic (18:1) and palmitic (16:0) acids are the most abundant both in backfat and IMF. Instead, the third quantitatively most important fatty acid in backfat is linoleic (18:2) acid, whereas in IMF it is stearic acid (18:0). These differences in fatty acid composition give backfat a more favorable desaturation ratio for 18:1/18:0 and better MUFA/SFA ratio as well.

## 3. Improving IMF Content and Composition through Selection

As stated above, fat content and composition are clearly dependent on the genetic background of each pig population. There are marked differences between breeds and even between lines within breed [16,17,18,19]. In general, differences across breeds and lines reveal that IMF and MUFA contents are inversely related to lean growth. In general, Duroc pigs show the best IMF:lean content ratio as compared to other breeds, thereby constituting the base of the majority of the genetic types used in markets where a relatively high level of IMF is demanded. The estimates of within-line genetic (co)variances indicate that there is enough genetic variation for IMF and MUFA to respond to selection. The heritability of IMF and MUFA is relatively high, around 0.4–0.7 [20,21]. Even though the genetic correlation of these traits with body weight is positive, reported estimates of their correlation with carcass lean content are negative. As a consequence, the continuous selection practiced for lean growth in the last decades has led to decrease the level of IMF and MUFA below the recommended for high-valued pork products. However, experimental evidence from commercial breeding programs indicate that this does not necessarily occur if the emphasis of selection is placed more in the growth rate rather than in the lean content [22,23]. In agreement with this, deterministic simulations have shown that there is room for a favorable correlated response in IMF when selecting for increased lean growth.

Experiments so far provide evidence that IMF responds to selection, but at the expense of increasing fatness, which is expected given the positive correlation that exists between both traits. For instance, after six generations of direct phenotypic selection on IMF, Schwab et al. [24] found that overall fatness increased in parallel to IMF. This line was used to identify signatures of selection associated with IMF. Although difficult to interpret, some of the signatures co-located with genes associated to overall fatness [25]. However, certain selection strategies can improve IMF [17] and MUFA [26] contents in parallel with lean growth. To date, two selection experiments have been undertaken in pigs to test whether IMF and lean growth can respond independently. Suzuki et al. [27] selected for seven generations a Duroc line for an index based on desired gains including body weight, backfat thickness and IMF. Results indicated that they were able to increase IMF while constraining, but not reducing, backfat thickness. The experiment in Ros-Freixedes et al. [28] was designed to test the opposite, whether backfat thickness can be reduced at restrained IMF, thereby assuming that IMF was already at the optimum value. In this experiment, consisting of three one-generation selection rounds on the mid-parent predicted breeding value for backfat thickness and IMF, selected pigs had greater lean weight but less IMF, although the decrease in IMF was half of the expected if it had not been restricted. Incomplete restriction of IMF was due to limitations in accuracy of breeding values and selection intensity. These results highlight the fact that it is feasible to manipulate IMF independently of lean growth in order to improve certain meat quality traits while maintaining the overall fatness of the carcass. However, this is a complex task that would benefit from the use of IMF-specific molecular markers to assist in the selection process.

There is little experimental evidence on the changes in fatty acid composition due to selection. In line with their correlation, Burkett et al. [29] found that selection for IMF increased MUFA and Ros-Freixedes [30] showed that selected pigs for MUFA tended to have more IMF. In this latter case, the response in MUFA fell short as compared to the expected because of limitations in selection intensity and accuracy of predicted breeding values. Successful selection for genetically opposite traits means applying a high selection pressure, which requires monitoring a large population. Selection accuracy can be improved either by increasing the phenotyping capacity or by using DNA markers. Several single molecular markers have already been described with relevant effects on IMF and MUFA (see Section 4.2). The integration of many markers into genomic selection has been shown to improve the accuracy of prediction of IMF [31] (see also Section 5 for a field example). However, because this type of selection is costly, the advantage achieved in genetic gain is not directly translated into economic benefits, particularly at low expenditure rates [32]. Interestingly, the use of genetic markers can have a greater impact on the selection for IMF and MUFA, either directly, if markers affect IMF or MUFA but not overall fatness, or indirectly, if markers affect also total fat content. In this latter case, the component of the polygenic effect that is independent of overall fatness can be better estimated.

## 4. The Molecular Basis of IMF Content and Composition

### 4.1. The Polygenic Structure Is Evidenced by Genome-Wide Association Studies

The development of molecular markers in the context of the PigMap project in the 1990s allowed conducting global analysis of genomic regions associated with complex quantitative traits such as meat quality-related traits. Measuring intramuscular fat composition is costly, which may explain the relatively low number of studies which have focused on this trait, compared to studies on intramuscular fat content. Early studies with low density marker panels already evidenced that IMF and MUFA are polygenic traits and that the chromosomal regions associated with them are very variable between breeds and even between muscles [33,34,35]. Despite this large variability, some regions were repeatedly found in QTL studies on IMF content or marbling score (reviewed in [36]). These regions include large areas of SSC1 (14 QTL), 4 (13 QTL), 6 (27 QTL) and X (10 QTL) (pigQTLdb, as of 17/08/2016). QTL maps on IMF composition reflect fewer coincidences between studies. Of relevance, QTL signals in SSC4, 8 and 14 have been frequently associated with SFA/MUFA relationship both in pigs of European and Asiatic origin. Most of the QTL found exhibit pleiotropic effects on several fatty acids, reflecting the enzymatic and transporting routes implicated in the fatty acid metabolisms. Genes underlying these common regions could be used in the selection programs of many commercial breeds. However, these QTL represent large chromosomal regions and finding the QTN, i.e., the causal mutation responsible for these effects, has proved to be ambitious and challenging. Among them, only two QTL at SSC6 and SSC14 have been translated into a possible QTN within a candidate positional gene [37,38,39].

The appearance of pig-specific high-density genotyping tools marked a turning point in the efficiency of genome scans. The use of these novel marker arrays reduced the genotyping time and improved the resolution of genome-wide association studies (GWAS). The large linkage disequilibrium rate in the pig genome allowed the definition of haploblocks and the selection of representative tag-SNPs in each one of them. Overall, results confirmed early observations, i.e., the breed- and muscle-dependence of the associated regions and the pleiotropic nature of the metabolism/transport pathways. The efforts from the Pig Genome Sequencing Consortia in assembling the pig genome materialized in a new and efficient tool in the form of genome browsers that can help to identify positional candidate genes underlying these relevant genomic regions. The latest GWAS studies in IMF composition have confirmed the relevance of regions in SSC4, 8 and 14 across most breeds and identified other breed-specific fragments [20,40,41]. On the whole, GWAS regions explain in most cases a relatively small fraction (<8%) of the phenotypic variation. The possibility to dive into the genome has allowed the identification of solid candidate genes; for instance, for the SSC4 region affecting PUFA content in muscle (*APOA2* gene, [42]) or for the SSC8 region for palmitic and palmitoleic acid composition (*ELOVL6*, [43,44]). Although several mutations and epigenetic marks have been found in these genes, causality has not yet been proven. This remains a constrain that needs to be tackled in the near future, as next-generation sequencing projects will need verification and validation of novel mutations at a high-throughput scale (see below).

Overall, there is a large overlap between genomic regions associated with IMF and fattening, particularly with backfat thickness. This reflects the positive genetic correlation that exists between IMF and backfat. In other words, mutations resulting on higher IMF content will also result in increased overall fatness. This limits the application of these markers in a pig breeding system that has lean content, among other traits, as a selection objective. Similarly, genomic regions associated with fat composition are mostly shared between muscle and fat traits [41], indicating that common fatty acid enzymatic/transport pathways take place in these two fat depots [45]. In terms of fatty acid composition this is not a limitation, as long as the lean content remains the same.

### 4.2. Molecular Markers Associated with IMF Content and Composition

Since the genetic correlation between IMF and backfat is not complete, there is the possibility to find markers that will improve IMF content without compromising the lean content of the carcass. Functional studies on adipocytes from different fat depots indicate that IMF cells have a delayed maturation and display differences in both metabolic and secretory functions [3]. Understanding how IMF physiology differs from other fat depots would facilitate developing strategies for genetic and non-genetic manipulation of IMF. It also opens up the possibility to find IMF biomarkers (for instance in blood [3,46]) or even DNA markers that would distinctly promote accumulation of fat in muscle or that would change the distribution of fat in the body (i.e., towards IMF at the expense of the carcass fatness).

In this regard, several markers have been reported in association with IMF content in pigs, particularly within functional or positional candidate genes. However, most of them are not specific for IMF. Instead, they promote overall fatness and, although an improvement in IMF content is achieved, this is counterbalanced by worse lean content. Many of these mutations have only been validated in a number of pig breeds and often they only explain a small percentage of genetic variation. In general, marker associations are influenced by the genetic background of the animals and magnitude of the observed effect are normally population specific. Three of the mutation with a more consistent effect on IMF content across studies are the melanocortin 4 receptor (*MC4R*), the (heart) fatty acid binding protein 3 (*FABP3*) and the leptin receptor (*LEPR*), although causality has not been proved for any of them.

A missense substitution in the exon 1 of the *MC4R* gene (c.1426A>G, p.Asp298Asn [47]) was one of the first polymorphisms to be linked to overall carcass fatness [48]. The MC4R is activated principally by α melanocyte stimulating hormone (MSH). It plays a central role in energy homeostasis and somatic growth by promoting satiety, energy expenditure and weight loss [49]. Several fat depots are affected by the c.1426A>G mutation, including IMF content, in Duroc [50,51] and Pietrain [52] but not in Duroc × Iberian pigs [53]. Similarly, a 5′UTR SNP polymorphism in the *FABP3* is also associated to fat deposition in muscle and carcass in Yorkshire [54] while mutations in the promoter, although only in Large White and not in Duroc and Piétrain, are specifically associated to IMF [55]. This partially agrees with the fact that *FABP3* gene expression correlates with IMF content [56,57]. Known as the heart-FABP, this gene is in fact expressed at high levels in skeletal muscle and kidney and at more moderate levels in many other tissues including adipose cells, where it participates in the intracellular uptake of long-chain fatty acids and their acyl-CoA esters. Likewise, the leptin receptor gene (*LEPR*) is active in many peripheral tissues. Besides the endocrine role in regulating food intake in the central neural system, leptin also exerts important paracrine and autocrine functions in muscle and fat cells regulating fatty acid uptake and oxidation. In pigs, a missense mutation in exon 14 of the *LEPR* (g.1987C>T) has been reported to affect overall fatness, in all breeds tested so far [20,39,57,58,59]. This is probably a combination effect of increased feed intake and lower fatty acid oxidation in peripheral tissues.

The link between muscular IMF and obesity-related genes is well reported [60,61]. In consequence, markers associated with IMF do not allow differential selection for IMF at restricted total fat content. The recent description of a c.2456C>A missense mutation in the *PCK1* gene has represented a major breakthrough [62]. In crossbred Duroc × Landrace/Large White pigs, this polymorphism was associated with enhanced IMF deposition (+20.4%) and reduced backfat thickness (−9.9%). PCK1 participates in the synthesis of phosphoenoyl pyruvate from oxaloacetate, a metabolic step at the crossroad of gluconeogenesis and fatty acid esterification. Further studies are needed to clarify the dual role of this enzyme in subcutaneous and IMF. If the results from Latorre et al. [62] are validated in other pig populations this will definitely promote this marker as a strong candidate to be integrated in marker-assisted selection programs of the pig industry.

Regarding IMF fatty acid composition, mutations in a small number of genes have been found to be related to variation in some of the fatty acid fractions. For instance, several promoter mutations in the ELOVL fatty acid elongase 6 (*ELOVL6*) gene have been associated with C16 (16:0 and 16:1) to C18 (18:0 and 18:1) content in *longissimus dorsi* muscle and backfat [43,44]. This enzyme elongates saturated and monounsaturated C12 to C18 fatty acids and it was a strong positional candidate gene for several compositional QTL at SSC8 in backfat [41,63] and muscle [40,64]. The mutations in the promoter have been linked to the disruption of several transcription factor binding sites and also to differential methylation of local CpG islands which correlate with gene expression. These changes in composition are not accompanied with variation in the fat content either in the muscle or in the backfat. The same research group has recently described a non-synonymous mutation in exon 4 of the apolipoprotein A2 (*APOA2*) gene associated with the content of several PUFA in muscle [42]. This gene encodes for the second most abundant protein of the high-density lipoprotein particles, and it is involved in facilitating lipid transport from peripheral tissues to the liver. Although it needs to be functionally validated, it co-localizes with a QTL for PUFA content in SSC4, 90–92 cM [40].

Oleic acid is the major MUFA in pork and represents 35%–45% of total fatty acid content although in acorn-fed Iberian pigs it can reach up to 55% of total IMF [65]. Oleic acid has been linked to consumer acceptability of high quality cured products, which represent a niche market of added value. Therefore, markers affecting oleic acid content are likely to have an impact in meat quality in general, and particularly in pricy traditional products. The stearoyl-CoA desaturase (SCD) enzyme is responsible of producing oleic and palmitoleic MUFA from stearic and palmitic SFA. A polymorphism in the *SCD* gene promoter has been associated with oleic content and desaturation index in fresh meat and dry-cured products [37]. Originally detected in Duroc pigs, the effect of the marker is stable across fattening time points, across muscles, and has been validated in Duroc lines and Duroc-sired crossbreds [37,66,67]. In our Duroc reference population this marker explains up to 27% of the genetic variance for MUFA content (up to 14.7% for oleic) [20]. This mutation is used as an example with field data on the possibilities of improving IMF composition in Section 5.

### 4.3. The Contribution of Functional and Massive Sequencing Data

Complex questions need to be tackled from more than one perspective. In the context of traits with a complex genetic background, structural genomic experiments (as seen in the last section) have been complemented with functional approaches, involving the study of the transcriptome of target tissues. The interest in comparing patterns of active and inactive genes between breeds differing in fattening (and growth) potential was developed from even before there were pig-specific microarrays (reviewed in [68]). However, the development of high-throughput techniques, such as cDNA and oligo-based arrays or high-throughput RNA sequencing (RNA-seq) approaches, represented a turning point in how genes are evaluated. Microarrays and RNA-seq represent valuable tools to study the transcriptome and its regulatory mechanisms at a global level. They give a quantitative snapshot of the activity of the cells in terms of RNA production. Initial microarray design tended to be tissue-specific, including only the genes that were active in each tissue. Soon, this strategy was dismissed as it became apparent that most genes were active in most tissues (although at tissue-specific levels). The latest annotation of the human genome indicates that there are 20,441 coding, 22,219 non-coding and 14,606 pseudogenes, producing a total of 198,002 different transcripts (http://www.ensembl.org/Homo_sapiens/Info/Annotation, accessed on 17 October 2016). Current annotation in pigs has identified similar number of protein-coding genes, but fewer non-coding transcripts than in humans but it is highly likely that, when the pig genome annotation is more advanced, similar numbers are encountered. In this regard, most porcine microarrays have been designed to target protein-coding genes, which limits the information that can be obtained from the RNA samples. In contrast, massive parallel sequencing methodologies are more flexible on defining the target. Library preparation protocols can be accommodated to include (or restrict) a wide variety of RNA populations. Moreover, RNA-seq has other additional advantages including the identification of novel transcript variants and the detection of sequence polymorphisms. Therefore, this technology gives currently the most comprehensive screening of key genes and processes participating in these complex phenotypes and, more importantly, it represents a more powerful approximation to detect population-specific markers associated with the traits.

A number of studies have compared RNA-seq transcriptome data from breeds with contrasting growing and fattening abilities. Most of these experiments compared lean, fast growing breeds such as Yorkshire or Landrace with indigenous Chinese or Korean breeds [69,70,71,72], or with European local breeds [73], which have an overall fattier phenotype. There is a common trend among these results, which is the overexpression in the leaner breeds of genes related to: (i) muscle differentiation and contraction along with changes in gene expression which promote; (ii) glucose production via gluconeogenesis and glycolysis; but also (iii) lipid catabolism; and (iv) mitochondria function. Although actual differentially expressed genes change between studies, these four main functions are common to most RNA-seq transcriptome experiments comparing lean to fat pig phenotypes, indicating that selection for lean growth has promoted rapid energy production in muscle. Similar results were obtained when comparing muscle transcriptomics from a Polish Landrace and Piétrain pigs [74]. Other studies have directed their attention to differences within a population. For instance, Puig-Oliveras et al. [75] chose two groups of pigs from an Iberian × Landrace population based on muscle fatty acid profiles. The authors linked the expression profile with the concentration of certain fatty acids, which cannot rule out that accumulation of certain fat molecules, such as PUFA, have affected expression. For instance, the well-known effect of PUFA in inhibiting genes associated to glucose uptake and lipogenesis can cause a feedback loop between muscle fatty acid composition and gene expression which, in turn, changes fatty acid composition.

Recent studies have shown that non-coding genes are crucial for adipocyte differentiation and function. Generally, non-coding RNAs (ncRNAs) can be sorted into housekeeping and regulatory ncRNAs. Housekeeping ncRNAs include transfer RNAs (tRNAs), ribosomal RNAs (rRNAs), small nuclear RNAs (snRNAs), small nucleolar RNAs (snoRNAs) and are usually expressed constitutively, while regulatory RNAs consists of piwi-interacting RNAs (piRNAs), microRNAs (miRNAs), and long non-coding RNAs (lncRNAs) [76]. Interaction between coding genes and miRNA in pigs differing in growth and fat deposition have been assessed and networks of integrated regulation have been built [71]. A catalogue of lncRNAs related to adipocyte differentiation is available in humans and mice, some of which have been validated by functional studies [77]. These lncRNAs have been found to participate in all the anabolic (adipogenesis and lipogenesis) and catabolic (lipolysis, fatty acid β-oxidation, and thermogenesis) routes involved in fat accumulation. One particular example is the *SRA* (steroid receptor RNA activator) gene which encodes the SRA protein but is also transcribed into an lncRNA which is a transcriptional coactivator of steroid and non-steroid receptors. During pre-adipocyte differentiation *SRA* expression is induced by 2-fold and it promotes differentiation partly via coactivation of *PPARγ* [78]. Thus far, no mutations in pig lncRNAs have been linked to IMF and fatty acid composition. However, these non-coding genes should be also considered when looking for markers for these polygenic complex traits.

Another novel area of research is the study of adipose-muscle cross-information by extracellular vesicles [79,80]. These mediate at least in part the paracrine effects of the cells by transferring proteins, lipids, and genetic material to target cells. In the context of adipose-muscle interaction, these vesicles represent a cross-cellular transport of regulatory information to modulate angiogenesis, adipogenesis and other cell pathways. For instance, they are particularly enriched in messenger RNA encoding for transcription factors that regulate these pathways, and also in miRNA that target these particular messenger RNAs. The role of transcription factors as master regulators of muscle and adipose functionality have also been highlighted by other groups [81,82], which has helped identifying key regulators of mesenchymal cell differentiation such as FOS, FOXO1 and NR3C1 and NR3C4.

From efforts from the human ENCODE project it has become evident that much of the genomic variation that affects phenotypes is not in coding regions (which represent approximately 1.5% of a mammalian genome) but in regulatory regions (which might account for up to 80% of the genome sequences). A network of as many as one million enhancers have been described in the human genome [83], which regulate the expression of coding and non-coding genes. Annotation of the pig genome is underway as part of the FAANG (Functional Annotation of Animal Genomes) international initiative [84]. As costs for whole-genome sequencing become more affordable, sequence variability at the whole-genome level is becoming an alternative to positional or physiological candidate gene approach. As an alternative to whole-genome sequencing, sequence variation information can also be retrieved from RNA-seq experiments and linked to expression levels [85]. The big limitation, and a severe bottleneck, still is how to perform large-scale functional tests on the SNP/INDEL/CNV mutations resulting from these experiments, particularly in non-coding regions. Among the proposed solution, imputation of functional data from human and model species into livestock sequences has succeeded in some recent works [86]. This is based on the fact that functionality is much better conserved among species than the actual sequence [87,88]. Other (experimental) alternatives are large-scale cell culture-based assays for SNP functional validation (Sequenom Inc., San Diego, CA, USA) and massively parallel reporter tiling assays, which have recently been used to test 4.6 million nucleotides spanning 15,000 putative human regulatory regions [89]. Functional validation of sequence variation remains a constraint that needs to be tackled in the near future, as next-generation sequencing is becoming more affordable.

## 5. The Use of Molecular Markers to Improve Meat Quality: An Example with Field Data

Including IMF and fatty acid composition in the selection objectives of breeding companies has several drawbacks, such as that the measurement is costly and usually unavailable on the selection candidates. Consequently, in traditional breeding schemes, the genetic evaluation for fatty acid content is more likely to be based on phenotypes of slaughtered littermates and other relatives of the selection candidates. Genotyping cost is one of the main factors limiting the implementation of genomic selection in pigs because, despite improving the prediction accuracy of the estimated breeding value (EBV), the value of any individual pig is lower and the generation interval is shorter than in cattle [90]. Therefore, the benefit of implementing genomic selection for IMF and MUFA must be assessed against the benefit that would be achieved by allocating economic resources to phenotyping a greater number of relatives of the selection candidates instead of genotyping them with SNP chips. The use of a smaller number of molecular markers that have a good predictive potential over these traits might represent a more affordable alternative. However, as stated in the previous section, identifying key molecular markers is not straightforward and might represent an overwhelming challenge.

Our research group has identified two markers (*SCD* and *LEPR*) with strong and consistent effects on IMF and fatty acid composition [20,37]. A polymorphism in the *SCD* promoter (AY487830:g.2228T>C; [37]) is associated with increased desaturation from SFA to MUFA in Duroc pigs. For gene *LEPR*, a polymorphism at exon 14 (NM_001024587:g.1987C>T; [39]) could be associated with feed intake regulation and indirectly affect IMF and fatty acid composition. These two markers explained a great percentage of genetic variance of IMF and fatty acid content traits (~15% to 30%, each) and could be used for marker-assisted selection [20].

We compared three different methods for genetic evaluation of IMF and fatty acid traits in our Duroc resource population. The first method was genomic prediction using a Bayes B model using high-throughput genotypic data, the second method was a traditional best linear unbiased prediction (BLUP) estimation without genotypic data, and the third method was BLUP with the two genetic markers at *SCD* and *LEPR*. The correlation between genomic EBV (GEBV) and the adjusted phenotypic values of the testing dataset was used as a measure of the prediction accuracy. The correlations are shown in Table 2. Note that these correlations should be divided by the square root of heritability of the trait to convert them to accuracies of GEBV as predictors of true breeding values.

### 5.1. Method A: Genomic Prediction Using Bayes B

We genotyped 135 purebred Duroc barrows using the PorcineSNP60 v2 Genotyping BeadChip (Illumina, CA, USA). After data quality control, the remaining data comprised 36,432 SNPs. We used 65 animals born in 2002–2003 as training data to estimate the SNP effects and then to predict the GEBV of the 70 animals born in 2009–2010. The substitution effect of each SNP was estimated using the Bayes B approach [91], which is described in detail in [20].

Genomic prediction by Bayes B using all SNPs available (scenario 36k, Table 2) had contrasting correlations: high (0.48–0.60) for SFA, MUFA, and 18:1/18:0 ratio; moderate (0.28) for 18:1; and low (0.04–0.10) for IMF, PUFA, and SFA/PUFA ratio. A GWAS with the same genotyped animals identified that the *SCD* and *LEPR* loci were the most influential on IMF and fatty acids in the population studied. Markers at *SCD* and *LEPR* were selected as representative of these two associations and genotyped independently. Then, both training and prediction were performed using only one or both of these two SNPs (scenario *SCD*/*LEPR*, Table 2). The correlations for the most poorly predicted traits rose to 0.36–0.48, with only a small penalty for the traits with the highest correlations (0.43–0.54). The rest of SNPs from the chip had very poor predictive capacity (0.03 to 0.17) probably due to the fact that small effect estimates are less reliable given the limited size of the training set.

### 5.2. Method B: BLUP without Genotypic Information

BLUP predictions were performed using a data set consists of 119,390 pedigree-connected pigs, from which 110,855 have at least one recorded trait but no genotypic data. In order to compare, we used the same 70 animals as in Method A (above) as testing set. The amount and quality of the phenotypic data greatly affected the accuracies achieved. In unitrait (U) models, only the studied trait was evaluated. In practice, traits such as body weight and backfat thickness are measured on routine performance tests at age of 180 days and they have large amounts of records that are used in genetic evaluations, including phenotypes for the selection candidates (around 110,000 records for each trait). In multitrait (M) models, IMF and the fatty acid compositional traits were evaluated conjointly with body weight and backfat thickness. All models used 936 records of IMF and fatty acids from pigs born from 2002 to 2007 (phenotypes with a low relationship degree), and some models additionally used 196 records from littermates of the testing set (L if included, NL if not).

Unitrait BLUP without genotypic information provided moderate correlations only when records from littermates were available, ranging from 0.31 to 0.39 for all traits except SFA and 18:1/18:0 ratio. With multitrait BLUP, great increments in correlation can be achieved for those traits that are more correlated to body weight and backfat thickness. For example, in our resource Duroc population, the genetic correlations of IMF with body weight and backfat thickness were 0.29 and 0.44, respectively, while 18:1/18:0 ratio did not improve because it was genetically uncorrelated to body weight and backfat thickness (genetic correlations were −0.07 and 0.00, respectively) (own unpublished data). The littermate phenotypes on IMF content and fatty acid composition became less beneficial for the multitrait model.

### 5.3. Method C: BLUP Accounting for SCD and LEPR Genotypes

Instead of genotyping a few animals with a high-density SNP chip (Method A), in Method C we genotyped a much larger group of animals for two SNPs with validated effect on IMF and fatty acid composition. We genotyped 915 pigs for AY487830:g.2228T>C (*SCD*) and 803 for NM_001024587:g.1987C>T (*LEPR*). In this case, the breeding value of each animal was calculated as the genotypic value of the *SCD* and *LEPR* markers plus a polygenic effect. This method provided the best accuracies. With univariate models and no records from littermates, accuracies from BLUP with genetic markers were similar to those predicted with only the *SCD* and *LEPR* markers. By adding records from littermates and on body weight and backfat thickness, the correlations were 0.47–0.48 for IMF and 18:1, 0.53–0.55 for MUFA and 18:1/18:0, and as high as 0.62–0.70 for SFA, PUFA, and SFA/PUFA.

Genetic markers resulted particularly beneficial for those traits poorly predicted with phenotypic data only. Thus, as compared to BLUP without genotypic data, the *SCD* and *LEPR* markers substantially improved the accuracy for SFA, MUFA, 18:1, and 18:1/18:0. Contrarily, these markers did not contribute much to increasing the prediction accuracy of IMF, PUFA, and SFA/PUFA.

### 5.4. Practical Implications

Overall, our results indicate that it is possible to obtain good prediction accuracies of breeding values of IMF and fatty acid traits based only on phenotypic records of close relatives and correlated performance traits. A breeding program involving IMF and MUFA should first focus on designing a system for recording IMF and MUFA at slaughter and then on developing a BLUP genetic evaluation procedure based on pedigree-connected phenotypes. For this, individual traceability of pigs from rearing to slaughter and the implementation of a feasible recording routine at the abattoir, are both needed. Fortunately, new non-destructive on-line equipment, mostly based on near infrared spectrometry (NIRS), is becoming available to cope with this need with very promising results [92].

Selected genetic markers, such as *SCD* and *LEPR* loci in our Duroc population, may enhance the accuracy of BLUP-based breeding values for some meat quality traits, especially when the phenotypic data available are insufficient for accurate predictions. If additional markers are incorporated, custom low-density SNP panels could be developed [93] with which to improve selection decisions at any stage of the breeding scheme.

Our results show no benefit of using high-density SNP panels over phenotypic data and singled-out genetic markers. However, we used a relatively small training set and it is possible that larger training sets allow for a more accurate estimation of marker effects and better predictions with the whole SNP chip, which in turn could be used for the prediction of other traits in the selection objectives. While genotyping costs are a concern, according to a simulation by Tribout et al. [32], only a small set of animals needs to be phenotyped and genotyped annually to create a training population suitable for implementing genomic selection for traits such as IMF and fatty acid composition, and, furthermore, there are strategies to reduce genotyping costs such as imputation from low-density marker arrays [94]. Focusing on a small panel of relevant markers, with large and consistent effects on the population, has the additional advantage that more pigs can be genotyped with the same budget.

## 6. Conclusions

Meat quality cannot be defined by a single meat attribute. However, IMF content and composition affect important quality attributes such as tenderness, juiciness, flavor, nutritional value, fat melting point and rancidity potential. Given the incomplete genetic correlation of IMF and fatty acid composition with total fat content, they can both be improved through selection independently from overall fatness. However, their measurement is costly and not easy, usually requiring determinations performed on meat samples taken at slaughter. Therefore, selection strategies would benefit from the use of molecular markers. Meat quality is a complex polygenic trait and, unfortunately, with one exception, no IMF-specific markers have been identified and only a few have been associated to fatty acid composition. However, as we develop a better understanding of how the genome works it is becoming clear that both coding and non-coding genes influence polygenic traits. Moreover, most of the genome has a regulatory function which makes identification of causal mutations even more difficult. However, it is possible to have good predictive ability of breeding values even with a small number of molecular markers as long as they explain a sufficient fraction of the genetic variance of the traits. As shown in our field data, this information can very effectively complement pedigree-based genetic predictions using phenotypic data from relatives. Altogether, this advocates for the development of population specific low-density marker arrays, particularly in populations with genotyping budget constraints.

## Figures and Tables

**Table 1:** Fatty acid composition (%, mean ± standard error) of backfat (BF), m. longissimus dorsi (LD) and m. gluteus medius (GM) in a purebred Duroc line ^1^.

Fatty Acid	BF	LD	GM
14:0	1.51 ± 0.02	1.51 ± 0.03	1.57 ± 0.02
16:0	21.75 ± 0.13 ^b^	25.45 ± 0.17 ^a^	25.51 ± 0.13 ^a^
16:1	2.34 ± 0.06 ^c^	3.69 ± 0.08 ^a^	3.39 ± 0.06 ^b^
18:0	10.28 ± 0.18 ^b^	12.78 ± 0.24 ^a^	12.59 ± 0.18 ^a^
18:1	46.69 ± 0.22 ^a^	45.74 ± 0.29 ^a,b^	45.41 ± 0.21 ^b^
18:2	14.14 ± 0.17 ^a^	7.22 ± 0.23 ^c^	8.36 ± 0.17 ^b^
18:3	1.02 ± 0.01 ^a^	0.34 ± 0.02 ^c^	0.46 ± 0.01 ^b^
20:0	0.15 ± 0.01 ^b^	0.19 ± 0.01 ^a^	0.15 ± 0.01 ^b^
20:1	0.99 ± 0.01 ^a^	0.73 ± 0.01 ^b^	0.73 ± 0.01 ^b^
20:2	0.77 ± 0.01 ^a^	0.33 ± 0.01 ^c^	0.39 ± 0.01 ^b^
20:4	0.32 ± 0.10 ^c^	1.97 ± 0.14 ^a^	1.38 ± 0.10 ^b^
SFA	33.70 ± 0.24 ^b^	39.90 ± 0.33 ^a^	39.84 ± 0.24 ^a^
MUFA	50.02 ± 0.24	49.93 ± 0.33	49.51 ± 0.24
PUFA	16.26 ± 0.26 ^a^	9.87 ± 0.35 ^b^	10.61 ± 0.26 ^b^
16:1/16:0	0.10 ± 0.01 ^b^	0.14 ± 0.01 ^a^	0.13 ± 0.01 ^a^
18:1/18:0	4.30 ± 0.04 ^a^	3.27 ± 0.05 ^b^	3.31 ± 0.04 ^b^
MUFA/SFA	1.49 ± 0.01 ^a^	1.25 ± 0.02 ^b^	1.24 ± 0.01 ^b^

^1^ This is a purebred Duroc line primarily used to produce high quality dry-cured ham. Data on fatty acid composition were obtained from 1380 barrows raised under commercial conditions with ad libitum access to a commercial diet and slaughtered at around 210 days of age (~125 kg of live weight). More detailed information on this population can be found in [16,17]. SFA, MUFA and PUFA are saturated, monounsaturated and polyunsaturated fatty acids, respectively. Within row, different superscripts indicate differences in means (*p* < 0.05).

**Table 2:** Correlations between predicted breeding values and adjusted phenotypes in a set of 70 pigs born in 2009 using different genomic pedigree and phenotypic data collected since 2002 (adapted from [6,20]).

Trait ^1^							
Method	IMF	SFA	MUFA	18:1	PUFA	18:1/18:0	SFA/PUFA
(A) Genomic prediction using Bayes B ^2^							
36 k	0.04	0.48	0.50	0.28	0.07	0.60	0.10
*LEPR*/*SCD*	0.46	0.48	0.43	0.36	0.49	0.54	0.47
Rest of chip	0.03	0.17	0.14	0.14	0.04	0.04	0.03
(B) BLUP ^3^							
U, NL	0.11	0.11	0.08	0.12	0.16	0.07	0.13
U, L	0.31	0.15	0.32	0.32	0.39	0.14	0.39
M, NL	0.41	0.39	0.30	0.29	0.61	0.08	0.60
M, L	0.42	0.41	0.40	0.38	0.67	0.15	0.67
(C) BLUP accounting for *SCD* and *LEPR* ^3^							
U, NL	0.34	0.50	0.39	0.31	0.41	0.51	0.41
U, L	0.42	0.52	0.51	0.44	0.51	0.53	0.51
M, NL	0.47	0.59	0.48	0.41	0.65	0.50	0.63
M, L	0.47	0.62	0.55	0.48	0.70	0.53	0.70

^1^ IMF: intramuscular fat; SFA: saturated fatty acids; MUFA: monounsaturated fatty acids; PUFA: polyunsaturated fatty acids; 18:1: oleic acid; 18:0: stearic acid. All traits determined in muscle gluteus medius; ^2^ 36k: prediction based on 36,432 SNP (PorcineSNP60 v2, Illumina, CA, USA), whose effect was estimated with Bayes B using 65 pigs born in 2002; *SCD*/*LEPR*: prediction based only on marker effects of AY487830:g.2228T>C (*SCD*), NM_001024587:g.1987C>T (*LEPR*), or both, estimated using 65 pigs born in 2002; ^3^ BLUP: best unbiased linear prediction based on full pedigree and data either only on the target trait (U, univariate model) or also on body weight and backfat thickness (M: multivariate model). BLUP models were solved including (L) or excluding (NL) the data on littermates, and the effect of genotypes at *SCD* and *LEPR* genes.
